# Efficiency in reducing air pollutants and healthcare expenditure in the Seoul Metropolitan City of South Korea

**DOI:** 10.1007/s11356-020-12122-y

**Published:** 2021-01-18

**Authors:** Subal C. Kumbhakar, Jiyeon An, Masoomeh Rashidghalam, Almas Heshmati

**Affiliations:** 1 Department of Economics, State University of New York, Binghamton, NY 13902 USA; 2 Department of Economics, Sogang University, 35 Baekbeom-ro (Sinsu-dong #1), Mapo-gu, Seoul, 04107 South Korea; 3 University of Tabriz, Tabriz, Iran; 4 Jönköping International Business School, Center for Entrepreneurship and Spatial Economics (CEnSE), Jönköping University, Room B5017, Gjuterigatan 5, P.O. Box 1026, SE-551 11 Jönköping, Sweden

**Keywords:** Air pollutants, Chronic illness, Healthcare expenditure, Cost efficiency, Stochastic frontier, Seoul metropolitan, South Korea, C23, H23, K32, O44, Q52, Q53

## Abstract

This study analyzes efficiency in the reduction of air pollutants and the associated healthcare costs using a stochastic frontier cost function panel data approach. For the empirical analysis, we use monthly data covering 25 districts in the Seoul metropolitan city of South Korea observed over the period January 2010 to December 2017. Our results show large variations in air pollution and healthcare costs across districts and over time and their efficiency in reducing air pollutants. The study concludes that efforts are needed to apply the World Health Organization’s air quality standards for designing and implementing location-specific customized policies for improving the level of air quality and its equal distribution, provision of health services, and improved efficiency in improving air quality standards. The study identifies a number of determinants of air pollutants and efficiency enhancement which provide useful pointers for policymakers for addressing the current environmental problems in South Korea.

## Introduction

Air pollution is a main contributor to diseases caused by the environment. Short- and long-term exposure to particulate matter (PM) and fine particles in the atmosphere adversely affect human health, reduce life expectancy, and lead to premature deaths (Adar et al. 2014; Weichenthal et al. 2014). PM consists of a complex combination of particles that increase the risk of respiratory and cardiovascular illnesses (Brown et al. 2013). It is also estimated that the mortality rate increases by 0.36% for 10 μg/m3 in PM_10_ concentration (Kim et al. 2019; Lu et al. 2015). And adverse conditions mean that people have to incur costs for doctors’ visits and pain leading to unnecessary additional costs. These also lower labor productivity, individual wages, and opportunities for career advancement all of which have a negative impact on both industrial and national output.

As reported by the World Health Organization (2014), in 2012 estimated premature deaths caused by air pollution was 7 million, which was equal to about 13% of the total number of deaths worldwide. And, in the 34 OECD (Organization for Economic Cooperation and Development) countries plus China and India, the healthcare costs of dealing with air pollutants were estimated at a combined total of 3.5 trillion dollars (US$) in 2010 (WHO Regional Office for Europe, OECD 2015). Evidence also shows that air pollution leads to costs running into several trillion dollars for societies each year.

Recently, South Korea has attracted attention because of a rapid increase in the concentration of fine dust in its atmosphere. Based on an OECD report, Korea will endure the most significant economic costs of outdoor air pollution among all the OECD countries by 2060 (OECD 2016). According to the OECD report, if no further action is taken to reduce air pollution, South Korea will face a considerable increase in human mortality rates from 359 to 1109 per million in 40 years. From an economic perspective, air pollution can increase a country’s economic burden because of increased morbidity and a corresponding increase in healthcare costs (Kim et al. 2019). Gyeonggi-do province, in which the Seoul metropolitan city is located nearby, in particular, has comparatively high PM pollution (Hien et al. 2018; Ustulin et al. 2018), and it has been incurring high environmental costs (Yoo et al. 2008). Hence, the need for an efficient and effective healthcare system is a major topic of discussion among social planners and policymakers in Seoul.

Healthcare spending in many OECD countries has been increasing dramatically over the past decade. More than 70% of the healthcare expenditure in these countries is financed through public healthcare provisions (Varabyova and Müller 2016). South Korea’s national healthcare costs continue to increase, but they are still low compared to the OECD average. However, its healthcare costs are growing at the fastest pace among the OECD countries. In 2012, South Korea spent 97.1 trillion won on national healthcare, accounting for 7.6% of its GDP, which is lower than the OECD average of 9.3%, but the annual average growth rate^1^ of healthcare cost is the highest in South Korea among OECD countries (OECD Health Data 2014).

An increase in healthcare expenditure has become a major problem for South Korea. By looking at the following figure (see Appendix Fig. 9), one can see that healthcare costs are on the rise. Not all cost increases can be attributed to air pollution, but healthcare expenditure from air pollution has been steadily increasing. The South Korean government also has a public healthcare system like OECD countries and subsidizes at least 40% of the total healthcare costs although this rate varies depending on the type of hospital and the classification of patients under Articles 44 and 19^2^ of the National Health Insurance Act. This is why many policymakers are particularly interested in evaluating the performance of different health systems and understanding how efficiently the resources invested in the healthcare sector are being used and if there is a possibility of improving the value of the invested money.

Hence, this study measures the technical cost efficiency of healthcare services in 25 districts in the Seoul metropolitan city. For quantifying healthcare’s cost efficiency frontier, we use a recent approach in the stochastic frontier analysis (SFA) that allows a simultaneous evaluation of the cost function’s parameters and factors shaping the districts’ efficiency. We use healthcare expenditure as the dependent variable and air pollutants’ composite index and number of patients as the key independent variables. Air pollutants include SO_2_, PM_10_, O_3_, NO_2_, and CO. Air pollutant-related chronic diseases include vascular mobility and allergic rhinitis, atopic dermatitis, asthma, and asthma persistence status. We estimate the flexible translog model as well as the restricted nested versions of the model including the Cobb-Douglas functional form.

To the best of our knowledge, there is no study on efficiency in air pollution’s effects on healthcare expenditure. We explored the stochastic frontier analysis to estimate not only overall technical healthcare cost efficiency but also efficiency of air quality that affects efficiency of health services or healthcare expenditure. This paper estimates that air quality induces an increase in healthcare expenditure, resulting in the (in)efficiency of health services. The study sheds light on efficiency in reducing air pollution on healthcare expenditure and the efficient-optimal allocation of resources invested in the healthcare sector.

The rest of this paper is organized as follows. The “Literature review” section reviews the literature, and the “Data” section describes the data, while the “Air pollution in South Korea” section discusses air pollution, air quality, and source variability in Korea. The “Model specification and estimation” section gives the model’s specifications and estimation. The “Estimation results and analysis” section gives the estimation results and their analysis, and the last section, the “Summary, conclusion, and implications of the results” section, gives the conclusion.

## Literature review

Improving the efficiency of public spending on healthcare is a priority across the globe and has been a subject of study for many years. The methods for estimating efficiency are non-parametric or parametric applied to a panel or cross section of countries, provinces, districts, and hospitals. Kocaman et al. (2012) evaluated the efficiency levels of healthcare systems in 34 OECD countries by applying the non-parametric data envelopment analysis (DEA) methodology. They used the number of hospital beds, number of physicians, health costs, and the ratio of health expenditure to GDP as input variables. The variables infant mortality rates and life expectancy were used as outputs. Their findings showed that 10 countries had efficient healthcare systems. Four years later, a study by Çetin and Bahce (2016) showed that 26 of the 34 OECD countries had efficient healthcare systems.

Using DEA for 30 European states, Asandului et al. (2014) evaluated the efficiency of their public healthcare systems. These authors used health adjusted life expectancy, life expectancy at birth, and infant mortality rate as output variables. The number of hospital beds, number of doctors, and public health expenditure as percentage of GDP were used as the input variables. Their results showed that most of the countries were inefficient when it came to dealing with air pollution-related illnesses.

Using the DEA methodology, Ahmed et al. (2019) estimated the technical efficiency of health systems in Asia and found that 91.3% (42 of 46) of the countries were inefficient in maintaining the technical efficiency of their health systems. They also found that countries in the high-income group (Japan, Singapore, and Cyprus) were more efficient than other countries. They used bed density, primary education completion rates, and population density as the determinants of efficiency. Using data from 173 countries and applying the DEA methodology, Sun et al. (2017) examined the performance of national health systems in these countries. According to their results, the national healthcare system’s efficiency was 78.9%, indicating a potential saving of 21.1% on health spending per capita. African and West Pacific countries had the lowest and highest efficiency of 67.0 and 86.0%, respectively. Tigga and Mishra (2015) assessed and compared the healthcare systems across states of India using the DEA methodology. They found that only six of the 27 states were technically fully efficient and had efficiency scores of 100.0. The rest of the states were technically inefficient and were using more than required inputs for achieving their levels of output.

In another study, Top et al. (2020) measured the healthcare system’s efficiency in 36 African countries and compared the efficiency levels of these countries using the DEA method. According to their results, 21 (58.33%) of the 36 African healthcare systems were efficient. Senegal was found to be inefficient in terms of input and output variables. The Gini coefficient and the number of nurses per 1000 patients had a positive and significant effect on the inefficiency levels of national healthcare systems. Ibrahim et al. (2019) estimated the technical efficiency and total factor productivity of sub-Saharan African (SSA) countries’ healthcare systems during 2010–2015. They found that healthcare systems in SSA were inefficient over the studied period. Kirigia (2015) identified weak healthcare leadership and management; inadequate manpower; and deficiencies in service delivery, vaccines, health financing, health informatics, and technology as factors leading to the inefficiency of African healthcare systems.

Grigoli and Kapsoli (2013) quantified the inefficiency of public health expenditure and estimated potential gains of improving efficiency in a sample of 80 developing economies over the period 2001–2010. The authors used the parametric stochastic frontier model to control for the socioeconomic determinants of health and for providing country-specific estimates. They found that African economies had the lowest efficiency among the countries studied. Herwartz and Schley (2018) used the SFA approach for studying how diversity and regional deprivation governed (in) efficiencies in the provision of healthcare services in districts in Germany. They established lower healthcare efficiencies in urban and higher healthcare efficiencies in rural areas.

In an extensive review, Worthington (2004) studied empirical techniques and selected applications in a frontier efficiency measurement of healthcare. He found that for-profit organizations were generally more efficient than their public-sector counterparts. Efficiency was also positively related to organizational size and healthcare organizations and industries’ efficiency improved over time.

Conversely, a significant volume of literature explains the nexus between air pollutants and healthcare expenditure. Martinez et al. (2018) show that air pollution led to significant economic costs for people in Macedonia’s capital Skopje. They found that long-term exposure to PM_2.5_ (49.2 μg/m3) led to an estimated 1199 premature deaths (confidence interval 95%, 821–1519) in 2012. They also found that the social cost of the predicted premature mortality rates due to air pollution were between 570 and 1470 million euros. Apergis et al. (2020) investigated the long-run dynamics of environmental pollution and healthcare expenditure across 178 countries during 1995–2017. Their results show that a 1% increase in national income and a 1% increase in CO_2_ emissions increased the health expenditure by 7.2% and 2.5%, respectively. They established that low-carbon emissions significantly reduced future healthcare expenses. Narayan and Narayan (2008) examined short- and long-run effects of environmental quality on healthcare expenditure in eight OECD countries. They reported a positive and significant effect of carbon emissions on health expenditure in the short run. However, they established that in the long run, sulfur oxide and carbon emissions had an inelastic and positive impact on health expenditure.

Studies such as those by Qureshi et al. (2015), Assadzadeh et al. (2014), and Apergis et al. (2018) examined the emissions-healthcare expenditure nexus across countries over different time periods. In South Korea, Yoo et al. (2008) examined the impact of environmental costs on morbidity, mortality, soiling damage, and poor visibility. Their study found that households’ monthly willingness to pay (WTP) for a 10% reduction in the concentration of major pollutants in Seoul was 5494 Korean won (US$ 4.6) and the total annual WTP for the entire population of Seoul was about 203.4 billion Korean won (US$ 169.5 million). An and Heshmati (2019) analyzed the effects of five air pollutants (SO_2_, CO, NO_2_, O_3_, and PM_10_) on healthcare expenditure in South Korea and found a statistically positive and significant association between some of them. Their findings showed that air pollution led to unnecessary costs including costs for doctors’ visits, pain, and opportunity costs incurred because of visits to the doctor. This also lowered labor productivity and earnings. Using the China’s Urban Household Survey (UHS) database, Yang and Zhang (2018) estimated the effect of exposure to air pollution on Chinese households’ healthcare expenditure. They concluded that a 1% increase in yearly exposure to PM_2.5_ corresponded to a 2.94% increase in households’ healthcare expenditure.

In conclusion, we find a positive relationship between air pollution and healthcare expenditure. This paper investigates efficiency of public health expenditure or health services in the studies that we reviewed. Most of the existing studies that measure the efficiency of healthcare spending use non-parametric techniques that do not control for a diverse set of factors that influence healthcare expenditure. They also combine random error effects with inefficiency effects. Existing research also considers internal factors that affect the efficiency of health services, bed density, the number of nurses, management, and leadership. However, this paper considers the external environmental factor of air pollution to study the efficiency of health services. To the best of our knowledge, there is no study on efficiency in air pollution’s effects on healthcare expenditure using SFA.

Hence, this study fills this gap in literature in several ways. First, we employ a parametric stochastic frontier approach which accommodates factors that affect efficiency and separate inefficiency and random error effects. Second, we use monthly data which captures the within-year seasonal variations in air pollutants and their healthcare costs. Existing studies use yearly data, but our paper uses monthly data because South Korea has four distinct seasons. Third, the sample consists of population in districts in the Seoul metropolitan city which is a mega city hosting about 20%^3^ of South Korea’s population. An efficient and effective healthcare system is a major topic of discussion among social planners and policymakers in Seoul. Finally, we identify and estimate the effects of various determinants of inefficiencies in healthcare costs.

## Data

**Map 1:**
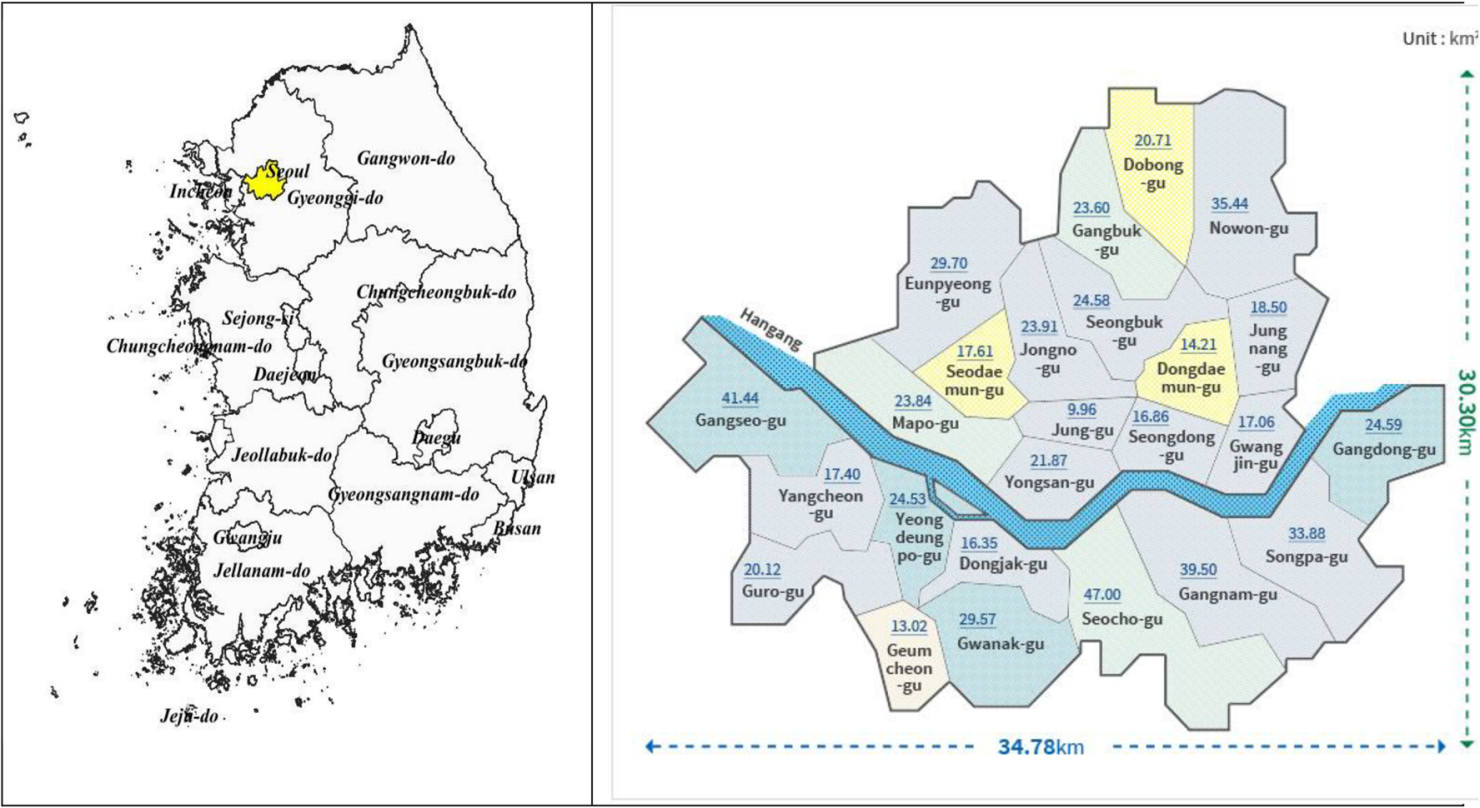
**A** and **B** Location of the Seoul Metropolitan city area and its 25 districts

For analyzing efficiency in reducing air pollutants’ effects on healthcare expenditure, this study compiled a monthly dataset during January 2010 to December 2017. The period is selected depending on data availability. The data contains information about concentration of air pollutants SO_2_, PM_10_, O_3_, NO_2_, and CO_;_ healthcare expenditure; number of patients; various districts’ socioeconomic characteristics including the number of cars, mining and industries operating in the districts, and the elderly; and atmospheric factors such as average temperature, wind speed, and monthly rainfall in the Seoul metropolitan city. The Seoul metropolitan city consists of 25 districts.^4^ Seoul’s location and its 25 districts are given in Map 1 A and B. All variables are at the monthly level and show the level and variations between districts in the Seoul metropolitan city.

Table 1 gives a brief description and the summary statistics of the variables, while Table 2 gives the Spearman correlation among the variables. The summary statistics given in Table 1 show that the average number of patients with particular diseases atopic dermatitis, vascular mobility and allergic rhinitis, asthma, and asthma persistence status, in Seoul’s districts, was about 9568 people with a minimum of 1880 and a maximum of 22,764 patients. Healthcare expenditure’s variables are measured monetarily in the South Korean currency, won. They are transformed to fixed values using the consumer price index (December 2017 prices). Per capita average healthcare expenditure was 444,493,800 Korean won. It varied across districts and over time in the interval of 95,040,000 and 1,034,706,000 South Korean won.

**Table 1:** Summary statistics of the data, 2010M_1_–2017M_12_ (2400 observations)

Variables	Description	Mean	Std. Dev.	Minimum	Maximum
Healthcare expenditure					
HCE	Per capita healthcare expenditure (HCE) adjusted by CPI (consumer price index)	444,493.80	170,513.80	95,040.00	1,034,706.00
Patients					
L20pat	Patients with atopic dermatitis	1096.35	426.86	285.00	2602.00
J30pat	Patients with vascular mobility and allergic rhinitis	6315.73	2818.09	803.00	16,168.00
J45pat	Patients with asthma	2058.29	905.77	352.00	6457.00
J46pat	Patients with asthma persistence status	97.29	89.95	1.00	731.00
Patient	L20pat + J30pat + J45pat + J46pat	9567.63	3792.16	1880.00	22,764.00
Composite air pollution index					
SO2	Concentration of SO2	0.005	0.001	0.002	0.011
PM10	Concentration of PM10	47.590	14.067	17.278	95.120
O3	Concentration of O3	0.020	0.009	0.005	0.051
NO2	Concentration of NO2	0.037	0.009	0.014	0.067
CO	Concentration of CO	0.574	0.150	0.191	1.179
COMP	Composite air pollution index	41.617	16.532	5.41e−06	100.000
Policy factors of inefficiency					
CARS	Cars enrolled in the districts	12,065,400	46,534	49,139	255,677
INDU	The number of mining and industries registered in districts	2355.33	1980.72	610.00	10,262.00
ELDER	The number of elderly (over 60 years of age)	46,322.47	12,829.21	15,852.00	76,442.00
Non-policy factors of inefficiency					
TEMP	Average temperature (°C)	12.85	10.22	− 8.80	29.20
WIND	Average wind speed (m/s)	1.70	0.44	0.10	3.30
RAIN	Monthly sum of rainfall	105.98	161.29	0.00	1116.00

1) Monetary unit is 1000 South Korean won 2) The data contains seasonal dummies

**Table 2:** Correlation coefficients of the variables (2400 observations)

	ln(HCE)	ln(Patients)	COMP	ln(CARS)	ln(INDUST)	ELDE	TEMP	WIND	RAIN
ln(HCE)	1.000								
ln(Patients)	0.990	1.000							
COMP	0.119	0.114	1.000						
ln(CARS)	0.795	0.773	0.054	1.000					
ln(INDU)	− 0.528	− 0.530	0.044	− 0.377	1.000				
ELDER	− 0.469	− 0.481	− 0.076	− 0.632	0.252	1.000			
TEMP	0.269	0.248	0.064	0.391	− 0.026	− 0.362	1.000		
WIND	− 0.405	− 0.397	0.110	− 0.347	0.576	0.195	0.048	1.000	
RAIN	0.341	0.343	− 0.035	0.320	− 0.177	− 0.270	0.226	− 0.029	1.000

The variables are divided into different groups. The first group relates to healthcare expenditure (dependent variable). The second and third groups form the two key independent variables and include the number of patients with diseases caused by air pollution. An increase in the number of patients or deterioration of air quality increases healthcare costs.

In this paper, healthcare expenditure is defined as total expenses or the sum of practiced costs and deductibles that are related to the treatment of particular diseases caused by air pollutants. This study elaborates on four different diseases that are likely to be a result of environmental deterioration: atopic dermatitis (disease code L20), vascular mobility and allergic rhinitis (J30), asthma (J45), and asthma persistence status (J46). Previous research concludes that these diseases are a result of environmental deterioration (Cho et al. 2013; Lee et al. 2016, 2018; Leem et al. 1998; Seo et al. 2000). The National Health Insurance Service has also designated these diseases as environmental diseases. Monthly healthcare expenditure (HCE) data is provided by the Health Insurance Review and Assessment Services.

The second group is related to the number of patients with health deficiency attributed to air pollutants in a given district. Table 1 shows the number of patients (PATIENT) with L20, J30, J45, and J46 diseases averaged by the districts. The data does not allow us to make a distinction between the four different diseases in the form of resource intensity requirements. Another reason for using their sum is for avoiding the multicollinearity problem.

The third group of variables is the five air pollutants: SO_2_, PM_10_, CO, O_3_, and NO_2_. Data on air pollutants is based on the hourly and is obtained from the National Institute of Environmental Research. The data consists of four atmospheric observations: suburban atmosphere, roadside atmosphere, national background concentration, and city atmosphere. It is measured by regional monitoring stations. The impact of individual air pollutants on healthcare expenditure differs. For instance, SO_2_ is released when fossil fuels such as coal and petroleum are burnt. This may cause temporary respiratory disturbances and exacerbate cardiovascular diseases. CO is formed when fuel with carbon is not burnt properly. It is mainly generated by the transport sector using fossil fuels. It reduces oxygen’s ability to be transported; exacerbates heart diseases, emphysema, and asthma; and reduces lung capacity. O_3_ is a secondary pollutant that is produced by photochemical reactions among nitrogen oxide, volatile organic compounds, and ultraviolet rays. Repeated exposure to ozone can cause lung damage, especially asthma and respiratory diseases. NO_2_ contributes to the formation of ozone and is formed during high-temperature combustion processes such as those in automobile and power plants. Large amounts of NO_2_ concentrations in the air lead to respiratory problems. PM_10_ is a fine dust that is discarded in the form of mixed particles in the air. It exacerbates respiratory diseases like asthma.

Since the air pollutants are correlated, we constructed a composite index to avoid the problem of multicollinearity. The composite index of air pollutants (COMP) is computed using the principal component analysis. The index is a weighted average of the principal components with eigenvalues greater than 1. The weights are the share of the total variance explained by each principal component.

The last group of variables include the districts’ characteristics such as the number of elderly (ELDER); registered and operated cars (CARS); the number of industries (INDUST); and atmospheric factors including temperature (TEMP), wind speed (WIND), and rainfall (RAIN). The variables’ trend representing technology is a combination of a year and a month (YMM).

Monthly demographic variables are obtained from Statistics Korea. The population demographic data is divided into 10 5-year age cohorts. This paper defines the elderly as those aged over 60 years. When national healthcare expenditure is divided by 10 age cohorts, it shows that the elderly had a large share in the total healthcare expenditure.

Data on the number of cars (CARS) and the number of industries (INDUST) registered in a district comes from Statistics Korea and the Ministry of Land, Infrastructure, and Transport. The number of cars is defined as the number of automobiles registered and operated in a given time period per capita inhabitants in the sample districts. The sum of mining and manufacturing industries is used as the number of industries. Emissions from automobiles and manufacturing processes can create secondary air pollutants which are formed when other primary pollutants emitted directly from a source react in the atmosphere. Therefore, cars and industries should be considered as key sources of air pollution and contributors to higher healthcare expenditures.

Atmospheric factors include monthly average temperature (TEMP), average wind speed (WIND), and average rainfall (RAIN) at the district level. These variables are included in the model’s specifications to capture their direct effects on air quality. The data is obtained from the Korea Meteorological Administration. These atmospheric variables influence the concentration of air pollutants and their effects on health and healthcare costs. We expect a positive trend effect of advancements in health technology on healthcare costs.

## Air pollution in South Korea

This section describes variations in air quality, healthcare expenditure, and the number of patients with four particular diseases across 25 districts in the Seoul metropolitan city over time. In particular, we look at recent conditions with respect to air quality and general patients and those who have diseases caused by air pollutants.

### Air pollutants and air quality standards

This study investigates the efficiency in reducing air pollutants and healthcare expenditure during January 2010 to December 2017. Air quality includes the concentration of five air pollutants: SO_2_, CO, NO_2_, O_3_, and PM_10_. Their concentration is measured in the hourly at specific monitoring stations. To avoid noise in the data and for capturing trends, the data is averaged into monthly observations for different districts.

Variations in the five air pollutants, healthcare costs, and the patient index across 25 districts are shown in Appendix Figs. 3, 4, 5, 6, 7, 8, and 9. These figures show that air pollutant levels varied over time and their development had seasonal fluctuations which depended on the intensity in energy use and weather conditions. In most of the districts, there was a decrease in the level of air pollutants over time. This decline was because of the revised Clean Air Conservation Act which came into force in 2015. In the press information by the Korean Center for Disease Control and Prevention (KCDC) on 29 February 2020, the word “social distancing” was used with reference to a collection of suggested activities, including keeping away from crowded areas, avoiding non-essential travel, and maintaining safe interpersonal distance. During February 2020, concentration of PM2.5 was decreased by 10.4% compared to an average rise of 23.7% over the same period in the preceding 5 years. CO and NO2 concentrations declined by 16.9 and 16.4% (Han et al. 2020).

SO_2_ had the highest value in Guro-gu and Gangseo-gu districts which have a concentration of large industrial complexes which do considerable fuel combustion and airports. PM_10_ is generated from diverse sources. As the figures in Appendix Figs. 3, 4, 5, 6, and 7 are shown, the 25 districts in the Seoul metropolitan city had bad air quality with respect to PM_10_. These districts had similar trends and indices. O_3_ had the highest value in Gangbuk-gu. NO_2_ is produced from high-temperature combustion processes such as those used in automobile and power plants. It had the highest value in Dongjak-gu and Seocho-gu which have highways nearby. CO is mostly emitted by the transportation sector. Gangnam-gu, Mapo-gu, Dongdaemun-gu, and Songpa-gu had the highest values of CO_2_ leading us to conclude that they are the most traffic-congested areas in Seoul.

Comparing Annual Korean Air Quality Standards, the variations in PM_10_ and NO_2_ exceeded the annual standards (Tables 3 and 4). The other pollutants were within the scope of the established standards.

**Table 3:** South Korea’s air quality standards

	1-h concentration	24-h concentration	Annual concentration
SO2	0.15 ppm	0.05 ppm	0.02 ppm
CO	25 ppm	9 ppm	9 ppm
O3	0.1 ppm	0.06 ppm	0.06 ppm
NO2	0.1 ppm	0.06 ppm	0.03 ppm
PM10	100μg/m^3^	100μg/m^3^	50μg/m^3^

Because CO and O3 do not have 24-h and annual standards available, the 8-h standard is used as the annual standard

**Table 4:** Air quality standards in other countries

	WHO annual standard concentration	Japan 24-h standard concentration	U.S. 1-h standard concentration
SO2	20μg/m^3^(24 h)	0.04 ppm	0.075 ppm
CO	–	10 ppm	35 ppm
O3	100μg/m^3^(8 h)	0.06 ppm(1 h)	0.075 ppm(8 h)
NO2	40μg/m^3^	0.04~0.06 ppm	0.1 ppm
PM10	20μg/m^3^	100μg/m^3^	150μg/m^3^

CO2 does not have the WHO standards available. SO2 and O3 also do not have annual standards available. The 24-h and 8-h standards are used as the annual standards. In Japan’s 24-h standards, O3 does not have 24-h standards. Therefore, the 1-h standards are used as the 24-h standards. In the US standards, O3 does not have 1-h , so the 8-h standards are used as the 1-h standards. One caveat is that PM10 contaminants must not exceed the average of 3 years more than once a year

Since the Korean air quality standards’ threshold is higher than the WHO’s annual air quality standards, many districts meet the Korean annual air quality standards but fail to satisfy the WHO’s higher annual air quality standards. A higher threshold means lower air quality. This shows that the Korean standards need to be revised to better protect the environment and the exposed people’s health. Table 3 and 4 compare the air quality standards recommended by the WHO and those practiced in Korea, Japan, and the USA. In general, the WHO’s standards are superior to those followed in these three countries.

These results show that each district has different value ranges for the five air pollutants and Korean’s air quality thresholds exceed the WHO’s annual air quality standards. Although the Seoul metropolitan city is small, environmental policies suitable for each district should be implemented because the districts have different characteristics and high population density. In South Korea, local governments rely on the central government for managing and controlling air quality. The local governments are not taking particular actions for improving air quality. However, the budget for managing air quality has recently been distributed to the local governments because the central government recognizes the necessity of reducing air pollution in the region. Earlier the central government managed most of the budget. Hence, this research concludes that it is necessary for local governments to come up with policies that strengthen air quality standards; they also need to control their budgets efficiently.

### Healthcare expenditure on environmental diseases

This study investigates trends in healthcare expenditure and the number of patients who have four environmental diseases. The variations in the 25 districts are shown in Appendix Fig. 8. Appendix Fig. 9 also shows that healthcare expenditure changed dramatically within and between seasons. It decreased in the summer and increased in spring and autumn. In contrast to the decline in air quality since 2015, variations in healthcare expenditure and the number of patients increased steadily. This means that the other factors affecting healthcare expenditure should be addressed when estimating the conditional relationship between healthcare expenditure and air pollutants.

Songpa-gu, Yangcheon-gu, Gangnam-gu, Gangseo-gu, Nowon-gu, Eunpyeong-gu, and Gwanak-gu had higher healthcare expenditures and number of patients. According to Seoul’s population statistics, these districts are densely populated and have the highest number of elderly people in Seoul. The severe air pollution in these districts affects the densely populated areas. Districts that are populated by high-income groups or the elderly have high healthcare expenditure and number of patients. If high-income groups live in a district, they invest in good health. They respond sensitively to their health. If the elderly live in such a district, they also spend more by visiting hospitals more often than the other age groups.

## Model specification and estimation

This study specifies the relation between healthcare expenditure and air pollutants. The objective of the districts in the Seoul metropolitan city is reducing air pollutants which will improve air quality thereby reducing healthcare expenditure. This study examines whether healthcare expenditure can be reduced and if so to what extent, conditional on controlling for the factors determining healthcare expenditure. We use the stochastic frontier (SF) cost function approach for this. The SF approach can estimate not only efficiency but also the factors that affect efficiency using a single-step approach. Therefore, we use this approach because this paper estimate whether air quality increases healthcare expenditure resulting in (in)efficiency in health services.

The SF cost model also allows us to check whether healthcare costs, given everything else, are at a minimum (which is often labeled as the cost frontier). If healthcare expenditure is above the cost frontier, the district is said to be inefficient or its cost efficiency is less than 100%. Cost inefficiency shows the percentage increase in costs or potential cost savings without reducing services. One can estimate this for each sample district and for each time period. We include factors that can explain the inefficiency component. Healthcare expenditure is estimated by including various control variables and the time trend. The SF model that we use is similar to that used in Badunenko and Kumbhakar (2017). The stochastic healthcare expenditure model is written as:

1 $$ \mathit{\ln}\mathrm{HC}{\mathrm{E}}_{it}=f\left({X}_{it};\beta \right)+{b}_i+{v}_{it}+{u}_{it}\left(Z{1}_{it},Z{2}_{it}\right) $$

where HCE is healthcare expenditure, subscripts *i* and *t* are individual districts and time periods, *X* is a vector of explanatory and control variables, *f*(.) is a parametric function, and *β* is a vector of unknown parameters to be estimated. We have two *X* variables: (1) number of patients with atopic dermatitis, asthma, vascular mobility, allergic rhinitis, and asthma persistence status, and (2) COMP is a composite air pollution index. In accounting for the multidimensional nature of the air pollution index, we apply a principal component analysis (PCA) for computing the COMP index.

The PCA methodology reduces the dimensionality of the dataset, while variability is preserved as much as possible (Hotelling 1933; Jolliffe and Cadima 2016). Principal components (PCs) are linear combinations of indicators in the initial dataset. Weights are assigned to the linear combinations of the original dataset defined as eigenvectors. The linear combination which describes the maximum variations is the first principal component. Additional components are generated sequentially, with each new component being independent of the previous ones. The indicators are strongly correlated within a principal component but the components are least correlated. Some key benefits of using PCA are the representation of complex multidimensional variables with fewer principle components (Wang and Wang 2015). Heshmati and Rashidghalam (2020) used the weighted average of principal components with eigenvalues greater than 1.

We use the share of the variance as weights in aggregating the principal components. This approach allows us to use the contributions of all indicators with eigenvectors greater than 0.30 in constructing the composite index. The signs and sizes of eigenvectors show their contribution to the overall index.

The error term is decomposed into three components—time-invariant district heterogeneity (*b*_*i*_), time-varying transitory inefficiency (*u*_it_), and the random error term (*v*_it_). Z1 and Z2 are vectors of policy and non-policy determinants of inefficiency. The empirical model assumes a flexible translog functional form incorporating non-linearity through squares and interactions of the *X* variables and the time trend. The flexible model’s specifications allow the elasticities to be district- and time-variant and the substitution and complementarity between the *X* variables and technical change to be captured.

The SF model used in Badunenko and Kumbhakar (2017) assumed that the inefficiency components were random. This resulted in a four-error components SF model. They made distributional assumptions on each of the error components and used the maximum likelihood method for estimating the parameters and predicting the inefficiency components. We do not make any distributional assumptions. The transitory component of cost inefficiency are specified as a deterministic function of its determinants (Kumbhakar et al. 2015; Paul and Shankar 2018):

2 $$ {u}_{it}\left(Z{1}_{it},Z{2}_{it}\right)=-\mathit{\ln}\Phi \left[{\xi}_0+{\sum}_m\left({\xi}_{1m}Z{1}_{mit}+{\xi}_{2m}Z{2}_{mit}\right)\right] $$

where *Ф* is normal cumulative distribution functions (CDF), *Ф* ≤ 1 implying ln *Ф* ≤ 0 and *u*_*it*_(*Z*1_*it*_, *Z*2_*it*_) =  −  *ln* Φ(.) ≥ 0. An advantage of this formulation is that no distributional assumptions are made about the inefficiency component. Instead of using normal CDF, one can use any other CDF (logistic) or other functions with the required properties. The downside of this formulation is that *u*_*it*_ is deterministic and works only when there are determinants. But there are no policy implications of inefficiency without determinants. The determinants provide a rationale for being inefficient. The district heterogeneity component *b*_*i*_ is assumed to be random with zero mean and constant variance (similar to random effects in a panel data model). The noise term *v*_*it*_ is assumed to have zero mean and constant variance. Since we use the non-linear least squares method, we do not assume any distribution for *b*_*i*_ and *v*_*it*_.

We assume *f*(.) to be represented by the translog function. The translog function with vectors *X*, Z1, Z2, and a time trend variable being added is specified as:

3 $$ \ln \mathrm{HC}{\mathrm{E}}_{it}={\beta}_0+{\sum}_j{\beta}_j{lnX}_{jit}+{\beta}_tt+1/2{\sum}_j{\beta}_j\mathit{\ln}{x}_{jit}^2+{\beta}_{tt}{t}^2+{\sum}_j{\beta}_{jt}{lnX}_{jit}t+{\sum}_{q=1}^3{Q}_q{D}_{qit}-\mathit{\ln}\Phi \left[{\xi}_0+\right.{\sum}_m\left({\xi}_{1m}Z{1}_{mit}+{\xi}_{2m}Z{2}_{mit}\right)+{v}_{it} $$

where *D* is a vector of seasonal dummies. The reference season is winter. It should be noted that the model in Eq. (3) is non-linear and it must be estimated by a non-linear estimation method. The estimated parameters are used for computing the elasticities of HCE with respect to X-variables and the rate of technical change as: *EX*_*jit*_ = *∂* ln HCE_*it*_/*∂* ln *X*_*jit*_and TC_*it*_ = *∂* ln HCE_*it*_/*∂t*. In addition, we also compute the estimated inefficiency ${\hat{u}}_{it}=-\mathit{\ln}\Phi \left[{\xi}_0+{\sum}_m\left({\xi}_{1m}Z{1}_{mit}+{\xi}_{2m}Z{2}_{mit}\right)\right]$ to obtain the estimates of the effects of the determinants of cost inefficiency from ${EZ}_{mit}=\partial {\hat{u}}_{it}/\partial {Z}_{mit}$.

## Estimation results and analysis

### Cost elasticities

The translog cost model in Eq. (3) is estimated using the non-linear least squares procedure in STATA. The restricted Cobb-Douglas form is also estimated. These models are nested. The estimation results are reported in Table 5. The fit of the two models measured as adjusted *R*^2^ is high at 0.98 and 0.99, respectively. The likelihood ratio test suggests that the determinants are jointly non-zero and translog is the preferred model’s specification. By adding squares and interactions of key variables to the flexible translog form (despite the constant parameters estimated) allows for non-linearity and estimating elasticities which are time and district variant. It avoids strong assumptions of constant responses in HCE to changes in the number of patients and air pollutant levels and changes in technology over time and across districts.

**Table 5:** Estimated stochastic frontiers and technical (In)efficiency effects (dependent variable—healthcare expenditure)

Variable	Cobb-Douglas		Translog	
	Coefficient	*t* statistics	Coefficient	*t* statistics
Frontier model				
Constant	4.195***	(166.57)	0.901	(0.40)
ln(Patient)	0.951***	(359.16)	1.454***	(3.84)
Comp air pollutants index	0.000664***	(6.40)	0.00119	(0.97)
T	0.000091***	(17.48)	0.00042	(0.89)
$$ \raisebox{1ex}{$1$}\!\left/ \!\raisebox{-1ex}{$2$}\right.\ln {\left(\mathrm{Patient}\right)}^2 $$	–	–	− 0.0371	(− 1.14)
$$ \raisebox{1ex}{$1$}\!\left/ \!\raisebox{-1ex}{$2$}\right.{\left(\mathrm{Comp}\right)}^2 $$	–	–	− 0.000022***	(− 4.11)
1/2 T^2^	–	–	0.0000015***	(18.74)
ln(Patient)*(Comp)	–	–	0.0000627	(0.48)
ln(Patient)*T	–	–	− 0.0000883*	(− 2.24)
(Comp)*T	–	–	0.0000001	(0.41)
D1 Spring	0.0208***	(5.77)	0.00167	(0.23)
D2 Summer	0.0351***	(10.29)	0.0174***	(3.43)
D3 Autumn	0.0416***	(10.19)	0.0264***	(6.89)
Policy factors of inefficiency				
Constant	–	–	− 1.544	(− 1.24)
ln(Patient)	–	–	0.455***	(6.02)
Ln(CARS)	–	–	− 0.157*	(− 2.40)
Ln(INDU)	–	–	0.00927	(1.73)
ELDER	–	–	− 0.299	(− 1.12)
T	–	–	0.00447***	(4.02)
Non-policy factors of inefficiency				
TEMP	–	–	− 0.0242*	(− 2.31)
WIND	–	–	0.0518*	(2.21)
RAIN	–	–	− 0.0145	(− 1.39)
Adj *R*^2^	0.9825	–	0.9913	–
Observations	2400	–	2400	–

*t* statistics in parentheses

^*^p < 0.05

^**^p < 0.01

^***^p < 0.001

All four basic coefficients of the restricted Cobb-Douglas cost model are as expected positive and statistically significant at less than the 1% level of significance. The coefficient of the number of patients is less than one suggesting increasing returns to scale. Healthcare costs increase in the aggregate quantity of air pollutants. The coefficient of the trend is positive suggesting a positive shift in the cost function or technical regress. The air pollutants phenomenon is relatively new and concerns about its health effects and general improved welfare and quality of health services may explain the positive shift in the cost function for a given number of patients.

The coefficients lnPatient and COMP represent the healthcare cost elasticities of patients and composite air pollutants in the Cobb-Douglas model. COMP is normalized, and as such it is interpreted like elasticity. These two variables significantly and positively affect healthcare expenditures. This finding is in line with previous studies like An and Heshmati (2019) who found that an increase in any of the three air pollutants (NO2, O3, and PM10) increased healthcare costs in South Korea. In their analysis, they found that differences in the amount of pollutants and related healthcare costs between the 16 cities and provinces in the country. These findings are also consistent with the findings published by the World Health Organization (2015) which addressed the economic costs of public health impacts of air pollution, with particular reference to the WHO’s European region countries. The report found the economic costs of air pollution and thus the benefits of cleaner air are very high in these countries.

The coefficient of time also shows that time positively and significantly affected healthcare expenditures. This may be because of the cost effects of intensity in using advanced health technologies. We also use dummy variables in the model to control for seasonal variations in the level of air pollutants. These dummies capture changes in healthcare costs resulting from any seasonal fluctuations attributed to seasonality in air pollutants. The reference quarter (D4) is winter. According to the Cobb-Douglas model, all the three quarterly dummy variables are positive and significant. The coefficient of Q_3_ is higher than the others, implying that, ceteris paribus, in the third quarter of the year, the healthcare expenditure is higher. Epidemiological studies show that healthcare expenditure and mortality are seasonally different. Mortality rates in older people have been found to increase during winter in Europe (Gemmell et al. 2000; Wilkinson et al. 2004), the USA (Fouillet et al. 2006), low and middle-income countries (Engelaer et al. 2014), and New Zealand (Davie et al. 2007). Other studies have shown that mortality rates increase in colder temperatures (Gasparrini et al. 2015).

Considering the translog model in Table 5, most of the coefficients are statistically significant.

We divide the determinants of cost inefficiency in the translog model’s specifications into policy-relevant and non-policy-relevant variables. The policy-relevant determinants can be influenced, while the non-policy-relevant determinants are exogenous and beyond the policymakers’ control. However, both categories affect air pollutants and their associated healthcare costs. We find that policy relevant variables such as CARS and industry are statistically significant. In the non-policy exogenous components of the determinants of inefficiency, TEMP and RAIN have significant and negative effects on cost inefficiency. These results go beyond another study in Seoul by Kim et al. (2014) which showed that atmospheric concentrations of PM10 and NO2 are lower in rainfall conditions compared to non-precipitation, and a noticeable difference in the mean concentration of PM10 was observed. One of the main mechanisms of precipitation washout is removal of airborne particulate contaminants. The variable WIND also has a positive and significant effect on cost inefficiency. High temperatures also contribute to poor air quality. During a heat wave, the intense heat and stagnant air increase the amount of zone emissions and particulate pollution. Increased use of cooling during a heat wave results in increased energy use and emissions.

We expected an increase in the number of cars and industries as a source of air pollutants and that a high share of elderly would contribute to districts’ inefficiencies in dealing with healthcare expenditure. However, the same increase in cost can also be caused by positive changes in advanced health technologies. We expected temperature and rain to have a positive effect on healthcare inefficiency, but we got an inconclusive effect of wind. Wind can influence the inflow and outflow of pollutants in a district. Thus, wind can reduce the concentration of air pollutants, but it also adds to the level sourced from neighboring regions. Detailed information about the elasticities of the determinants of cost inefficiency are presented in Table 6.

**Table 6:** Healthcare cost elasticities and estimated efficiency (TE)

		Mean	Std. dev.	Minimum	Maximum
Input elasticities and TC:					
Cobb-Douglas	Patient	0.95000	–	–	–
	Comp	0.00060	–	–	–
	TC	0.00001	–	–	–
Translog	Patient	1.07988	0.02634	1.0151	1.16623
	Comp	0.00087	0.00037	− 0.0005	0.00182
	TC	0.00029	0.00034	− 0.0003	0.00094
Elasticities of policy inefficiency’s determinants					
Patient		0.1830	0.0411	− 0.3544	− 0.0990
CARS		− 0.0630	0.0142	0.0341	0.1221
INDU		0.0037	0.0008	− 0.0072	− 0.0020
ELDE		− 0.1202	0.0270	0.0650	0.2327
T		0.0018	0.0004	− 0.0035	− 0.0010
Elasticities of non-policy inefficiency’s determinants					
TEMP		− 0.0097	0.0022	0.0053	0.0189
WIND		0.0208	0.0047	− 0.0403	− 0.0113
RAIN		− 0.0058	0.0013	0.0032	0.0113
Technical efficiency TE from the translog model					
TE		0.7643	0.0611	0.5123	0.8861

The average elasticities of cost with respect to the number of patients and air pollutants and rate of technical change computed based on the translog estimation results are very close to those obtained using the Cobb-Douglas method (see Table 6). According to Table 6, the elasticity of Patient and COMP variables is 1.08 and 0.0008, respectively. Since COMP is a normalized index, this means that for a 10-point increase in COMP the healthcare costs increase by 8%.

In terms of the magnitude of elasticities, the variable Patient is an important determinant of healthcare expenditure. In the specification of technical efficiency’s effects, elasticities of CARS and ELDER are about − 0.0630 and − 0.1202, respectively. Elasticities of INDU are 0.0037, which means that technical inefficiency increases in high productive and industrialized districts.

Table 6 also provides a summary of the estimated efficiency. The sample mean efficiency is 0.764 which means that there is scope for improving healthcare cost efficiency by 23.6%. The range of average district healthcare cost efficiency varies between 0.512 and 0.886. Despite applications of a similar environment and health policies in the Seoul metropolitan city area, the variations between the districts are relatively large.

### District heterogeneity and dynamics of efficiency

Variations in the overall cost efficiency and its components are presented in Table 7. In this table, districts are ranked based on their overall cost efficiency and location in relation to Han River. The mean cost efficiency varied across districts in the interval of 0.8125 (for Eunpyeong-gu) and 0.6562 (for Jung-gu). We did not find any systematic differences in efficiency between the northern (14 districts) and southern (11 districts) parts of Seoul separated by the river. Yet, the districts are differently exposed to air pollutants’ sources from the airport, wind flows, traffic flows, and population concentration.

**Table 7:** District-wise and year-wise estimates of mean efficiency (TE), using the translog model

Rank	District	TE	Location	Year	TE
1	Eunpyeong-gu	0.8125	North	2010	0.7096
2	Songpa-gu	0.8117	South	2011	0.7288
3	Nowon-gu	0.8092	North	2012	0.7413
4	Gwanak-gu	0.8044	South	2013	0.7571
5	Seongbuk-gu	0.7999	North	2014	0.7754
6	Gangseo-gu	0.7943	South	2015	0.7817
7	Yangcheon-gu	0.7851	South	2016	0.8032
8	Mapo-gu	0.7833	North	2017	0.8174
9	Guro-gu	0.7813	South		
10	Jungnang-gu	0.7804	North		
11	Gangnam-gu	0.7792	South		
12	Gangdong-gu	0.7791	South		
13	Seodaemun-gu	0.7745	North		
14	Gangbuk-gu	0.7713	North		
15	Dongdaemun-gu	0.7699	North		
16	Dongjak-gu	0.7672	South		
17	Seocho-gu	0.7642	South		
18	Gwangjin-gu	0.7608	North		
19	Dobong-gu	0.7511	North		
20	Yeongdeungpo-gu	0.7510	South		
21	Seongdong-gu	0.7484	North		
22	Jongno-gu	0.6955	North		
23	Yongsan-gu	0.6946	North		
24	Geumcheon-gu	0.6828	South		
25	Jung-gu	0.6563	North		

Location refers to the parts north and south of Han River (see Map 1B)

Developing overall cost efficiency over time is also given in Table 7. The mean cost efficiency increased from 0.7096 in 2010 to 0.8174 in 2017. This increased efficiency as a result of decreased air pollutants was a result of cost tightening environmental standards which applied to all locations, yet their effects may differ due to differences in initial time-invariant or slowly changing conditions in the districts.

**Fig. 1:**
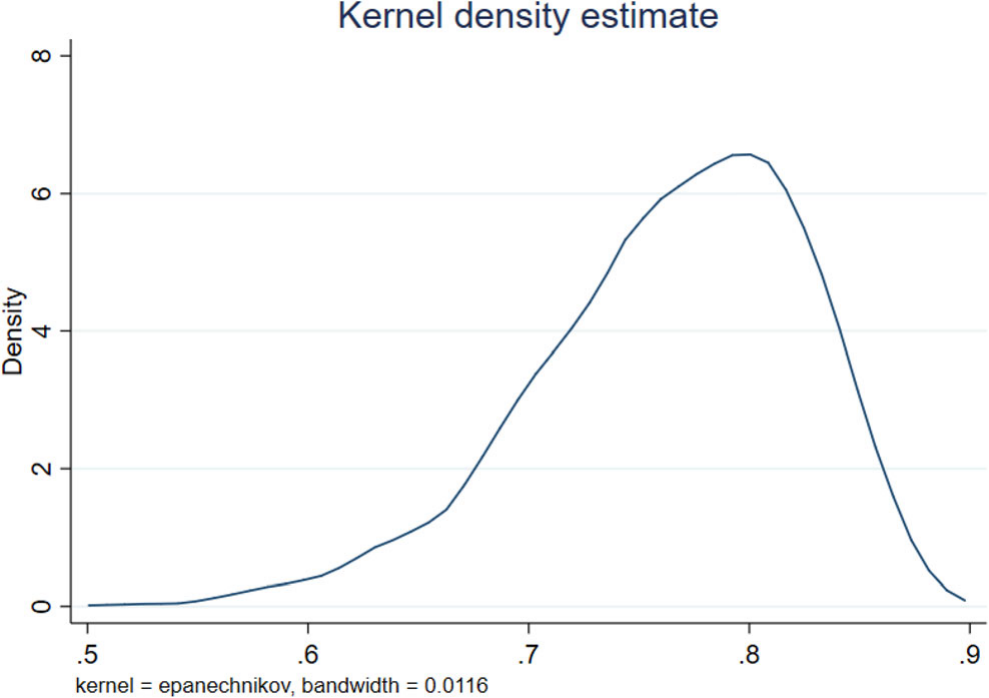
Technical efficiency distribution

**Fig. 2:**
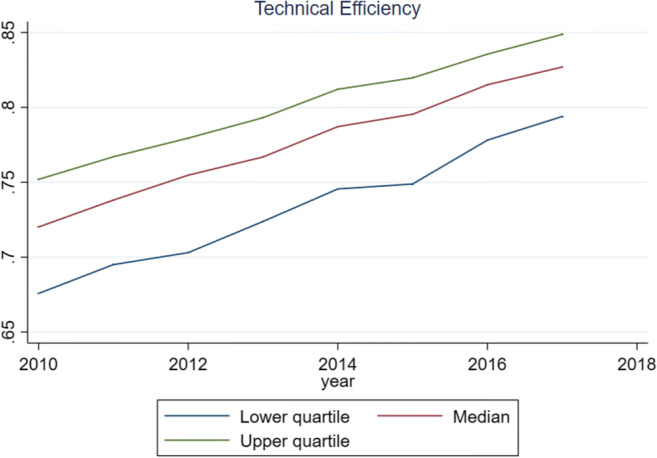
The median, first, and third quartile values of technical efficiency

The Kernel densities of the technical efficiency are given in Fig. 1. Technical efficiency is right skewed (see also Table 7). The median, first, and third quartile values of technical efficiency and its components are given in Fig. 2. In this figure, all the three lines develop in parallel and positively over time.

This study found evidence of significant variations in technical cost efficiency among the districts in the Seoul metropolitan city. Our results show significant district heterogeneity in performance and potential to reduce healthcare costs. Here our focus is on costs induced by air pollutants. Air pollutants are generated locally or regionally. The regional component can be better managed through cooperation between the districts while local air pollutants can be managed through the central government’s policies as also policies at the region level. City planning, regulations, and various incentives can reduce the level of air pollutants and shift from polluting to cleaner technologies. An integrated environment and joint provision of services implies close and inclusive participation of the districts in environmental policy formulations and their implementation.

Future research is encouraged to focus on improved definitions and measurement of air pollutants, patients, healthcare costs, identifying key determinants of cost and its efficiency, and equality of air quality across conjoint districts in Seoul. Seoul enjoys very high per capita incomes and hosts more than about 20% of South Korea’s population and is a key business center in East Asia. Efficiency and cost reductions should account for quality of life and well-being of the population and protection of the environment.

## Summary, conclusion, and implications of the results

There is evidence that air pollutants lead to adverse chronic illnesses primarily in major urban and densely populated areas. These chronic illnesses require medical treatment which means additional healthcare costs, reduced productivity, and economic burden. They influence health, labor supply, production, businesses, and travel and tourism negatively. This study analyzed the Seoul metropolitan city’s districts’ efficiency in reducing air pollutants and their associated healthcare costs. It estimated a stochastic frontier cost function approach with a three-error components structure. The empirical analysis was based on monthly balanced panel data covering 25 districts in the Seoul metropolitan city observed over the period January 2010 to December 2017.

Our results show that despite the geographic concentration of the sample districts, there is evidence of large variations in air pollution and healthcare costs across districts and over time. These relatively large variations are a result of the districts’ conditions and ability to effectively reduce air pollutants. The conditions can differ in the form of population density, wind flows, concentration of industries, and closeness to airports and highways. Efforts need to be made to apply the World Health Organization’s air quality standards which have lower thresholds than applying the national standards in the districts as they have higher thresholds. National standards with higher thresholds were designed considering lower per capita income in the past and for supporting the export-oriented business sector to be efficient, profitable, and competitive internationally. Seoul hosts more than about 20% of South Korea’s population with very high productivity and per capita incomes. The city can afford to invest more in higher living standards and quality. Such investments can be self-financed as their economic benefits are very likely to be more than the costs incurred.

The central and local governments should jointly design and implement both national and location-specific customized policies for improving air quality and its equality, provision of health services, and improving efficiency in enhancing the nationally determined air quality standards. This study identifies a number of determinants of air pollutants and efficiency enhancement practices providing useful pointers for policymakers for addressing the current environmental problems in Seoul in particular as it has a large share of the country’s population. Public investments and incentive programs are necessary for promoting private sector investments in development and use of clean technologies. South Korea is an industrially developed economy with high living standards, advanced human capital, a capacity for technology development, disciplined and high productive labor, and efficient governance. It can reallocate resources oriented towards improving the quality of life and the environment.

In addition, to reduce the burden of healthcare spending, the government should improve environmental governance, pay attention to the heterogeneity of healthcare costs in various districts in Seoul, and enhance the health of elderly people. Clean air policies such as the Seoul Metropolitan Area 2nd Air Management Strategy will reduce the burden of healthcare costs for the South Koreans. Further studies should investigate a closer collaboration between public health and environmental policies. In this study, we investigated vascular mobility and allergic rhinitis, atopic dermatitis, asthma, and asthma persistence status. In addition, air pollution can also play a role in a number of illnesses such as neonatal diseases and impairments in neuropsychology. This is an issue for future research to explore.

To improve the efficiency of healthcare provision and health outcomes within a district, resource allocation in Seoul should take regional deprivation and diversity into account. We suggest that Seoul’s healthcare-related policies should be responsible for the unbalanced healthcare efficiencies of different districts to some extent. In Jongno-gu, Yongsan-gu, Geumcheon-gu, and Jung-gu, the cost efficiency is low, and the government needs to adjust these areas’ industrial structures, environmental pollution rectification measures, disease prevention, and better treatment of diseases. We also recommend increasing the number of input and output variables in the healthcare system’s efficiency analyses.

## Figures and Tables

**Fig. 3:**
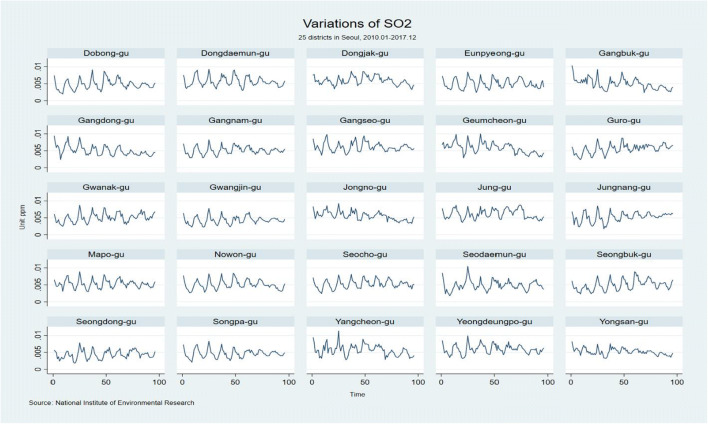
Variations of SO2 across 25 districts (January 2010–December 2017)

**Fig. 4:**
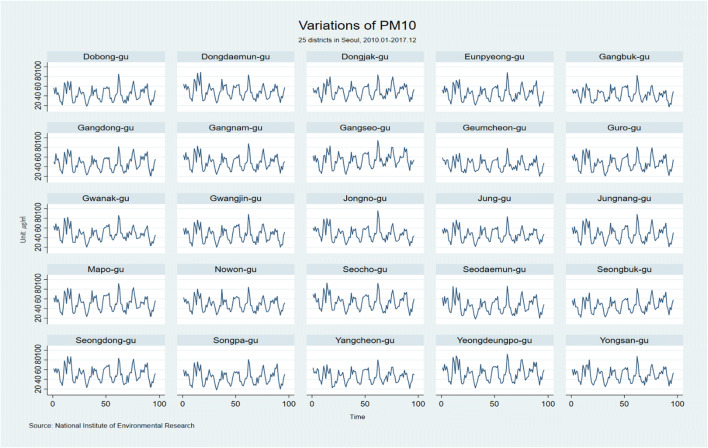
Variations of PM10 across 25 districts (January 2010–December 2017)

**Fig. 5:**
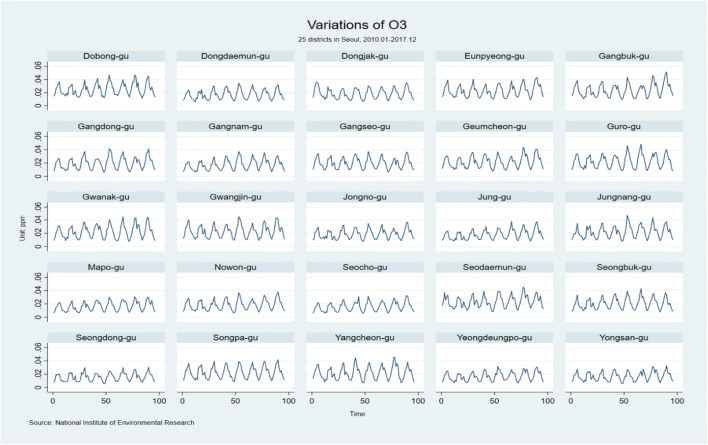
Variations of O3 across 25 districts (January 2010–December 2017)

**Fig. 6:**
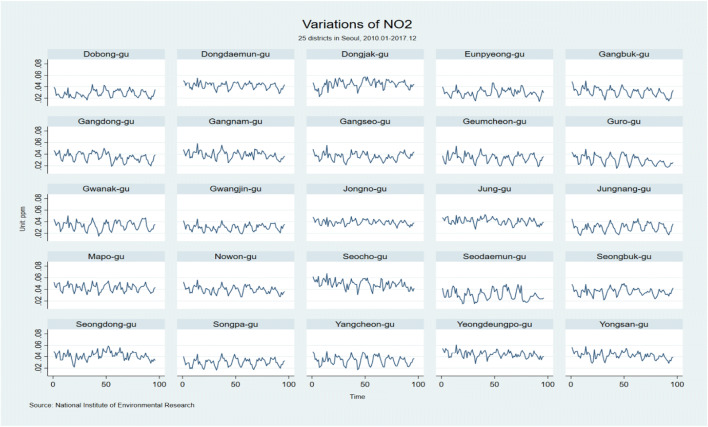
Variations of NO2 across 25 districts (January 2010–-December 2017)

**Fig. 7:**
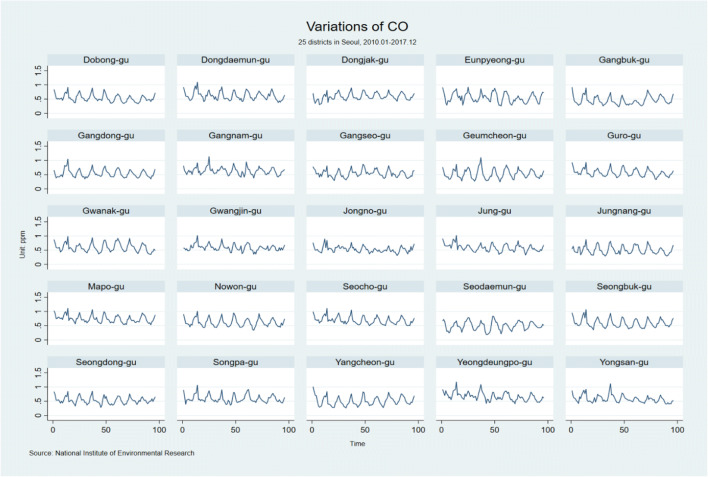
Variations of CO across 25 districts (January 2010–December 2017)

**Fig. 8:**
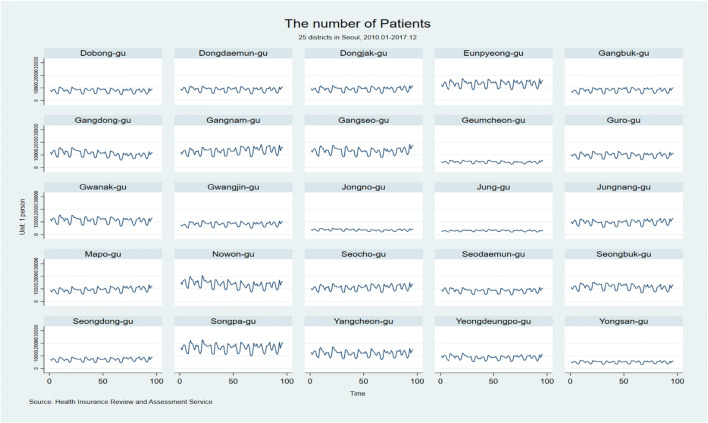
The number of patients across 25 districts (January 2010–December 2017)

**Fig. 9:**
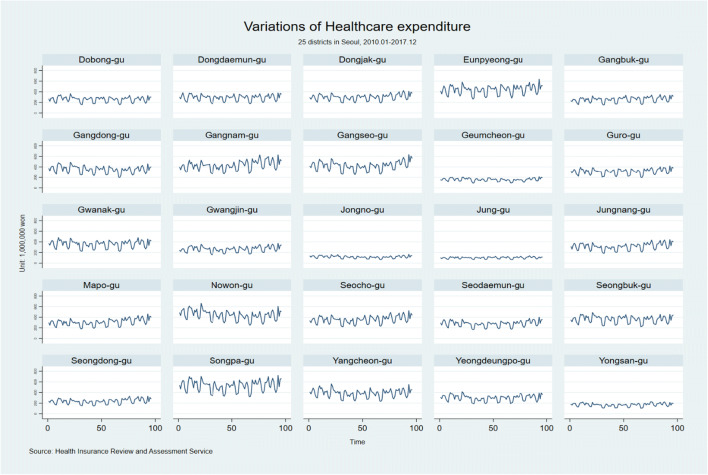
Variations of healthcare expenditure across 25 districts (January 2010–December 2017)

## Data Availability

The data and codes are available upon request.

## Footnotes

1 The real growth rate of South Korea’s national healthcare costs in the period 2007–2012 was 6.6%, while the corresponding costs for OECD countries were 2.3% during the same period.

2 The burden ratio and the amount of expenses to be borne by an individual are separately determined, but the expenses borne by medical institutions are calculated by considering the amount purchased by the medical institution as medical care benefits within the upper limit (Article 19 of the Enforcement Decree of the National Health Insurance Act).

3 Based on the metropolitan area (Seoul metropolitan city, Incheon metropolitan city, and Gyeonggi provinces), it accounts for 50% of South Korea’s population.

4 The Seoul metropolitan city’s 25 districts are Gangnam-gu, Gangdong-gu, Gangbuk-gu, Gangseo-gu, Gwanak-gu, Gwangjin-gu, Guro-gu, Geumcheon-gu, Nowon-gu, Dobong-gu, Dongdaemun-gu, Dongjak-gu, Mapo-gu, Seodaemun-gu, Seocho-gu, Seongdong-gu, Seongbuk-gu, Songpa-gu, Yangcheon-gu, Yeongdeungpo-gu, Yongsan-gu, Eunpyeong-gu, Jongno-gu, Jung-gu, and Jungnang-gu.
